# Malignancy as a Predictor and Potential Modifier of Laboratory Biomarker Prognostic Value in Acute Pulmonary Embolism

**DOI:** 10.3390/diagnostics16132130

**Published:** 2026-07-07

**Authors:** Sonja Salinger, Aleksandra Kozic, Stefan Ilic, Boris Dzudovic, Bojana Subotic, Jovan Matijasevic, Marija Benic, Tamara Kovacevic Preradovic, Ana Kovacevic-Kuzmanovic, Irena Mitevska, Vladimir Miloradovic, Ema Jevtic, Aleksandar Neskovic, Slobodan Obradovic

**Affiliations:** 1 Clinic of Cardiology, Clinical Center Nis, 18000 Nis, Serbia; sonja.salinger@gmail.com; 2 Faculty of Medicine, University of Nis, 18000 Nis, Serbia; 3 Medical Faculty of the Military Medical Academy, University of Defense, 11000 Belgrade, Serbia; a.djajic@gmail.com (A.K.); dzuda1977@gmail.com (B.D.); sloba.d.obradovic@gmail.com (S.O.); 4 Clinic of Emergency Internal Medicine, Military Medical Academy, 11000 Belgrade, Serbia; 5 Clinic of Cardiology, Military Medical Academy of Belgrade, 11000 Belgrade, Serbia; bojana.su@gmail.com; 6 Institute of Pulmonary Diseases Vojvodina, 21000 Novi Sad, Serbia; jovanmat99@yahoo.com (J.M.); marija7benic@gmail.com (M.B.); 7 School of Medicine, University of Novi Sad, 21137 Novi Sad, Serbia; 8 Clinic of Cardiology, Clinical Center Banja Luka, 78000 Banja Luka, Bosnia and Herzegovina; tamara.kovacevic@medicolaser.info; 9 School of Medicine, University of Banja Luka, 78000 Banja Luka, Bosnia and Herzegovina; 10 General Hospital Pancevo, 26000 Pancevo, Serbia; anakuzman@gmail.com; 11 Intensive Care Unit, University Cardiology Clinic, 1000 Skopje, North Macedonia; peovskai@yahoo.com; 12 Clinic of Cardiology, Clinical Center Kragujevac, 34000 Kragujevac, Serbia; vanja.miloradovic@gmail.com (V.M.); ema.jevtic@gmail.com (E.J.); 13 School of Medicine, University of Kragujevac, 34000 Kragujevac, Serbia; 14 Clinic of Cardiology, University Clinical Center Zemun, 11080 Belgrade, Serbia; neskovic@hotmail.com; 15 School of Medicine, University of Belgrade, 11000 Belgrade, Serbia

**Keywords:** pulmonary embolism, malignancy, biomarkers, risk stratification

## Abstract

**Background/Objectives:** Acute pulmonary embolism (PE) is a major cause of cardiovascular mortality, with prognosis influenced by hemodynamic status, comorbidities, and biomarker profiles. Although several laboratory markers have demonstrated prognostic relevance in PE, it remains unclear whether their predictive performance differs in patients with active malignancy. This study aimed to identify laboratory predictors of in-hospital mortality in acute PE and evaluate the modifying effect of malignancy on biomarker-based prognostic stratification. **Methods:** This retrospective multicenter cohort study included 2803 consecutive patients with confirmed acute PE enrolled in the Regional Pulmonary Embolism Registry (REPER) between January 2015 and April 2026. Univariate and multivariable logistic regression analyses were performed to identify predictors of in-hospital mortality in the overall cohort and subgroups stratified by malignancy status. Interaction analyses were used to formally assess effect modification by malignancy. **Results:** Active malignancy was present in 14.02% of patients, and overall in-hospital mortality was 11.10%. Multivariable analysis identified malignancy, CRP, glucose, creatinine clearance (CrCl), platelet count, and ESC risk category as independent predictors of in-hospital mortality. In-hospital mortality was significantly higher in patients with malignancy compared with those without (16.54% vs. 10.21%, *p* < 0.001). In the malignant subgroup, CRP and glucose remained independent predictors, whereas in non-malignant patients, CRP, glucose, CrCl, and ESC risk category were independently associated with outcome. Significant interactions between malignancy status and CrCl, age, glucose, and total leukocyte count suggest that the prognostic contribution of these variables may differ according to cancer status. **Conclusions:** Active malignancy is an independent predictor of in-hospital mortality in acute PE and appears to be associated with a more severe presentation. Our findings suggest that malignancy may also modify the prognostic performance of certain biomarkers. These observations suggest that conventional risk stratification tools may require cautious, malignancy-aware interpretation and that prospective studies validating malignancy-adapted prognostic frameworks are warranted.

## 1. Introduction

Acute pulmonary embolism (PE) is one of the most important cardiovascular emergencies in contemporary clinical practice. It continues to impose a substantial burden on healthcare systems globally, with reported in-hospital mortality ranging from approximately 2% in low-risk patients to over 30% among those with overt hemodynamic compromise [1,2,3].

Validated clinical scores—most notably the Pulmonary Embolism Severity Index (PESI) and its simplified version (sPESI)—provide a structured approach to early mortality risk assessment; however, their discriminatory performance may vary across real-world patient populations. Moreover, because they rely predominantly on clinical variables and do not incorporate routinely available biomarkers, additional prognostic information may be obtained from complementary biomarker assessment [4,5].

Clinical and laboratory biomarkers are gaining increasing importance as tools for prognostic assessment as they reflect key pathophysiological processes, including systemic inflammation, thrombotic burden, and cardiorenal dysfunction. Among these, C-reactive protein (CRP)—an acute-phase reactant reflecting interleukin–6–mediated systemic inflammatory activation—has emerged as a promising prognostic biomarker in acute PE [6,7]. Elevated CRP levels in acute PE have been associated with right ventricular dysfunction, greater thrombus burden, and increased short-term mortality, providing information complementary to conventional cardiac biomarkers [6]. D-dimer, as a direct marker of fibrinolytic activation and thrombotic burden, has been associated with adverse outcomes across different clinical presentations of PE [4]. Admission blood glucose, reflecting stress-induced catecholamine release, endothelial dysfunction, and glycation-related procoagulant effects, has been associated with adverse in-hospital outcomes in patients with acute PE [8,9]. Similarly, renal function, estimated using creatinine-based equations, may reflect reduced cardiorenal reserve in patients with acute PE [4].

Among clinical factors influencing PE prognosis, active malignancy has consistently been linked to adverse outcomes. Active cancer is associated with a three-to fourfold increase in short-term mortality after PE. In addition, large studies consistently show that in-hospital mortality is about twice as high in patients with malignancy compared with those without, while the overall risk of venous thromboembolism in oncological patients is estimated to be four– to sevenfold higher than in the general population. This elevated risk likely reflects a complex interplay of mechanisms, including tumor-driven overexpression of tissue factor, chronic systemic inflammation mediated by tumor-derived cytokines, endothelial injury, venous stasis, prothrombotic effects of antineoplastic therapies, and paraneoplastic activation of the coagulation cascade [10,11,12,13,14]. This bidirectional relationship, whereby thrombosis may promote tumor progression while malignancy sustains a hypercoagulable state, suggests that cancer-associated PE may differ biologically and clinically from PE occurring in patients without malignancy [12].

Whether routinely available laboratory biomarkers provide comparable prognostic information in patients with and without active malignancy remains insufficiently explored. Because malignancy may influence inflammatory activity, coagulation, renal function, and hematologic parameters, it could also affect the prognostic interpretation of these biomarkers. Therefore, the present study evaluated the association between routinely available laboratory parameters and in-hospital mortality in a large cohort of patients with acute PE, with particular emphasis on whether malignancy modifies these associations.

### Aim

The aim of this study was to evaluate the prognostic performance of routinely available laboratory parameters for predicting in-hospital mortality in patients with acute pulmonary embolism, with particular emphasis on the potential modifying effect of active malignancy. In addition, we compared biomarker-outcome associations between patients with and without malignancy to determine whether their prognostic performance differed according to cancer status.

## 2. Materials and Methods

### 2.1. Study Design and Population

This was a retrospective, multicenter cohort study based on data from the Regional Pulmonary Embolism Registry (REPER), established in 2015. The registry collects information on hospitalized patients diagnosed with PE confirmed by multi-detector computed tomography pulmonary angiography (MDCT-PA). Participating centers include five university hospitals in Serbia (Military Medical Academy Belgrade, Institute for Pulmonary Diseases Vojvodina, Clinical Centers Zemun, Niš, and Kragujevac), one general hospital (Pančevo), and three university cardiology clinics in Banja Luka (Bosnia and Herzegovina), Podgorica (Montenegro), and Skopje (North Macedonia).

For the present analysis, 2803 consecutive patients enrolled between January 2015 and April 2026 were included. The inclusion criteria were: age ≥ 18 years, symptoms potentially attributable to acute PE within the preceding two weeks, hospitalization in cardiology or pulmonology wards at the time of diagnosis, and positive MDCT-PA findings demonstrating at least one segmental or three or more subsegmental thrombi. Patients with only one or two isolated subsegmental filling defects (due to limited clinical relevance and diagnostic variability) and those admitted because of terminal illness were excluded.

Routine admission laboratory tests included complete blood count parameters (total leukocyte count (TLC), hemoglobin (Hb), platelet count (PLT)), while D-dimer and glucose were measured separately, using standardized methods across participating centers. Troponin, B-type natriuretic peptide (BNP), and CRP were measured within the first 24 h after admission, according to local laboratory protocols. Renal function was estimated by the Cockcroft-Gault formula retrospectively for all patients using serum creatinine values obtained at hospital admission. In subsequent analyses, values obtained by this formula were considered as CrCl. Active malignancy was defined as a PE occurring as the first clinical manifestation of cancer, or a PE in patients with a known cancer diagnosis who had received cancer-directed treatment within the preceding 6 months (including surgery, chemotherapy, immunotherapy, radiotherapy, or symptomatic oncological therapy).

### 2.2. Handling of Missing Data

Missing data were systematically assessed for all variables. The total study cohort comprised 2803 patients. However, the number of available observations varied across parameters: CRP (*n* = 2691), BNP (*n* = 882), Troponin I (*n* = 1139), D-dimer (*n* = 2597), glucose (*n* = 2718), CrCl (*n* = 2181), platelet count (*n* = 2729), total leukocyte count (*n* = 2779), neutrophils (*n* = 1720), lymphocytes (*n* = 1538), and hemoglobin (*n* = 2778).

For univariate analyses, all patients with available data for the respective variable were included. In the multivariable logistic regression model, only patients with complete data across all selected predictors were retained (listwise deletion), resulting in a final sample size of 1878. BNP was excluded from the multivariable model due to a high proportion of missing values (≈68.5%), which could have biased the analysis.

### 2.3. Outcome Measure

The primary endpoint was in-hospital mortality from any cause. As a secondary objective, we compared the predictive performance of routinely available laboratory markers between patients with and without active malignancy, with the aim of determining whether malignancy alters their prognostic value for in-hospital mortality in a real-world clinical practice setting.

### 2.4. Statistical Analysis

Continuous variables are presented as mean ± standard deviation or median with interquartile range (IQR), depending on data distribution, while categorical variables are reported as counts and percentages. Between-group comparisons (patients with vs. without malignancy) used Student’s t-test or Mann-Whitney U test for continuous variables and the chi-square test for categorical variables, as appropriate. Variables with skewed distributions were log-transformed (natural log) prior to regression analyses.

Associations with in-hospital mortality were first evaluated using univariable logistic regression. Variables that reached statistical significance (*p* < 0.05) in the overall cohort were considered candidates for the multivariable model, with the exception of BNP, which was excluded due to a high proportion of missing values (≈68.5%). Patients with complete data across all selected predictors were retained (listwise deletion), yielding a final sample size of 1878. The multivariable model was constructed using a forward stepwise likelihood ratio method.

For subgroup analyses (patients with and without active malignancy), univariable regression was performed for all predefined parameters, and variables that showed statistical significance in at least one subgroup were jointly entered into the multivariable models for both groups to ensure consistent evaluation of predictors across cohorts.

To further explore potential threshold effects and non-linear associations, biomarker values that emerged as independent predictors in the multivariable models were categorized into quartiles; each quartile was compared with Q1 as the reference. Quartile-based analyses were performed separately in malignant and non-malignant subgroups.

Interaction analyses were performed to evaluate whether the prognostic effect of biomarkers tested in univariable analyses on in-hospital mortality was modified by malignancy status. This approach was chosen because univariable analyses indicated that certain predictors were significant in one subgroup (malignant or non-malignant) but not in the other. By formally testing interaction terms (variable × malignancy), we aimed to determine whether the presence of malignancy accounted for these differences in predictive value.

Finally, receiver operating characteristic (ROC) analyses were conducted to assess the discriminatory performance of individual biomarkers in both subgroups.

Results are reported as odds ratios (ORs) with 95% confidence intervals (CIs) for regression analyses. A two-sided *p*-value < 0.05 was considered statistically significant. Statistical analyses were conducted using SPSS software (version 26, IBM Corp., Armonk, NY, USA).

## 3. Results

A total of 2803 patients with pulmonary embolism (PE) were included in the study. The mean age of the overall cohort was 64.78 ± 15.63 years, and 47.09% of patients were male. According to PTE risk stratification (according to ESC guidelines 2019), 795 (28.36%) patients were classified as low-risk, 703 (25.08%) as intermediate-low-risk, 871 (31.07%) as intermediate-high-risk, and 434 (15.48%) as high-risk PE. A total of 393 (14.02%) patients in our cohort were diagnosed with active malignancy. The overall in-hospital mortality rate was 11.10%, of which 55.63% was confirmed to be directly related to pulmonary embolism. Baseline characteristics are presented in Table 1.

### 3.1. Predictors of In-Hospital Mortality in the Overall Cohort

In univariate logistic regression analysis for in-hospital mortality, age, malignancy, and PTE risk category were significantly associated with the outcome. Among laboratory parameters, CRP, BNP, D-dimer, glucose, and TLC were positively associated with mortality. Conversely, higher CrCl, Hb, and PLT were associated with lower mortality risk. Troponin, Neutrophils, Lymphocytes, Hematocrit, and gender were not significant predictors. In multivariable analysis (BNP was excluded due to a high proportion of missing data), CRP, glucose, CrCl, PLT, PTE risk category, and malignancy remained independent predictors of in-hospital mortality (Table 2).

### 3.2. Comparison Between Malignant and Non-Malignant PE

For this analysis, variables that were included in the univariate regression model were compared between patients with and without malignancy. Patients with malignancy were significantly older compared to those without (66.78 ± 11.85 vs. 64.47 ± 16.13 years, *p* = 0.001), while the proportion of male patients did not differ significantly (43.8% vs. 47.6%, *p* = 0.159).

Significant differences were observed in several laboratory parameters. Patients with malignancy had higher CRP levels, D-dimer, and TLC, as well as lower lymphocyte count, Hb, hematocrit, and CrCl (CrCl borderline significant, *p* = 0.051). Platelet counts were also higher in patients with malignancy, although this difference was borderline significant (*p* = 0.050). The distribution of PTE risk categories differed significantly between groups (*p* < 0.001), with a notably higher proportion of patients with malignancy classified as high risk. No significant differences were observed in BNP, troponin I, neutrophils, or glucose between the groups (Table 3).

In-hospital mortality was significantly higher in patients with malignancy compared to those without (16.54% (65/393) vs. 10.21% (246/2410), *p* < 0.001). Among deaths, a smaller proportion of fatalities in the malignancy group were directly attributable to PE compared with the non-malignancy group (PE-related deaths: 28/65, 43.1% vs. 146/246, 59.3%).

### 3.3. Predictors of Mortality Stratified by Malignancy Status

In patients without malignancy, age, CRP, BNP, D-dimer, glucose, CrCl, Hb, TLC count, and ESC risk category were significantly associated with mortality.

In contrast, in patients with malignancy, fewer variables remained significant predictors. CRP, BNP, D-dimer, PLT, glucose, and ESC risk category were significantly associated with mortality, while age, CrCl, Hb, and TLC were not significant predictors.

In patients with malignancy (*N* = 261), multivariable analysis identified CRP and glucose as independent predictors of in-hospital mortality. In contrast, in patients without malignancy (*N* = 1617), CRP, glucose, CrCl, and ESC risk category remained independently associated with mortality (Table 4).

**Figure 1:**
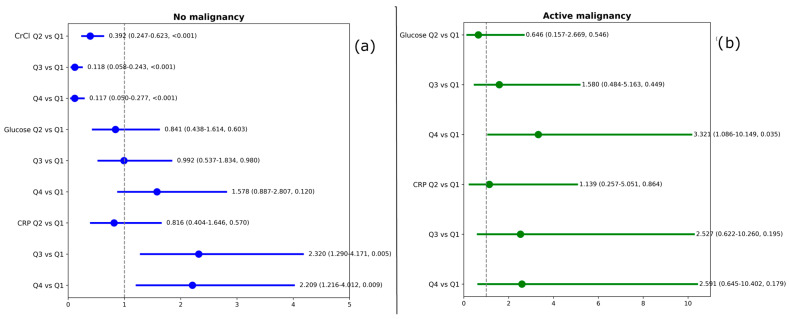
Quartile-based associations of biomarkers with in-hospital mortality according to malignancy status. Dotted vertical line indicates the null value (OR = 1). (**a**) Forest plot of odds ratios for CRP, glucose, and CrCl quartiles in patients without malignancy (*N* = 1617). Higher CRP quartiles (Q3 and Q4 vs. Q1) were significantly associated with increased mortality, while progressively higher CrCl quartiles (vs. Q1) were associated with significantly lower mortality risk. (**b**) Forest plot of odds ratios for CRP and glucose quartiles in patients with active malignancy (*N* = 261). Only the highest glucose quartile (Q4 vs. Q1) showed a significant association with mortality, while elevated odds ratios for CRP did not reach statistical significance versus Q1.

Quartile-based analyses compared each quartile with the reference Q1 and revealed different patterns of association between biomarkers and in-hospital mortality according to malignancy status. In patients without malignancy, higher CRP quartiles (Q3 and Q4) were significantly associated with increased mortality, while progressively higher CrCl quartiles were associated with significantly lower mortality risk. In patients with active malignancy, only the highest glucose quartile (Q4) showed a significant association with mortality. Although elevated odds ratios were also observed across CRP and D-dimer quartiles in malignant patients, these associations did not reach statistical significance when compared with Q1 (Figure 1).

### 3.4. Interaction Analyses

**Figure 2:**
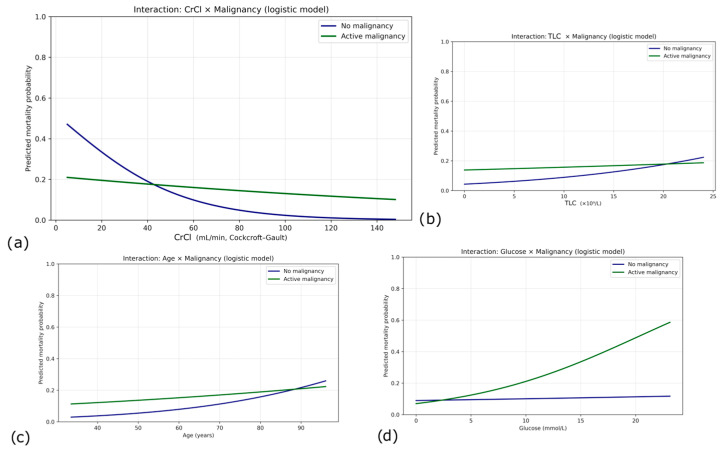
Interaction analyses of biomarkers with malignancy status in relation to in-hospital mortality. (**a**) Interaction between CrCl and malignancy status. (**b**) Interaction between TLC and malignancy status. (**c**) Interaction between age and malignancy status. (**d**) Interaction between glucose and malignancy status.

Interaction analyses were performed to assess whether the effect of selected variables on mortality differed according to malignancy status. A significant interaction was observed between CrCl and malignancy (OR for interaction 1.033, 95% CI 1.021–1.045, *p* < 0.001), indicating that the association between renal function and mortality differed between groups. Similarly, significant interactions were observed for age (OR 0.975, 95% CI 0.950–1.000, *p* = 0.050), glucose (OR 1.120, 95% CI 1.039–1.208, *p* = 0.003), and TLC (OR 0.940, 95% CI 0.911–0.969, *p* < 0.001) (Figure 2).

In contrast, no significant interaction effects were found for CRP, D-dimer, Hb, or platelet count.

**Figure 3:**
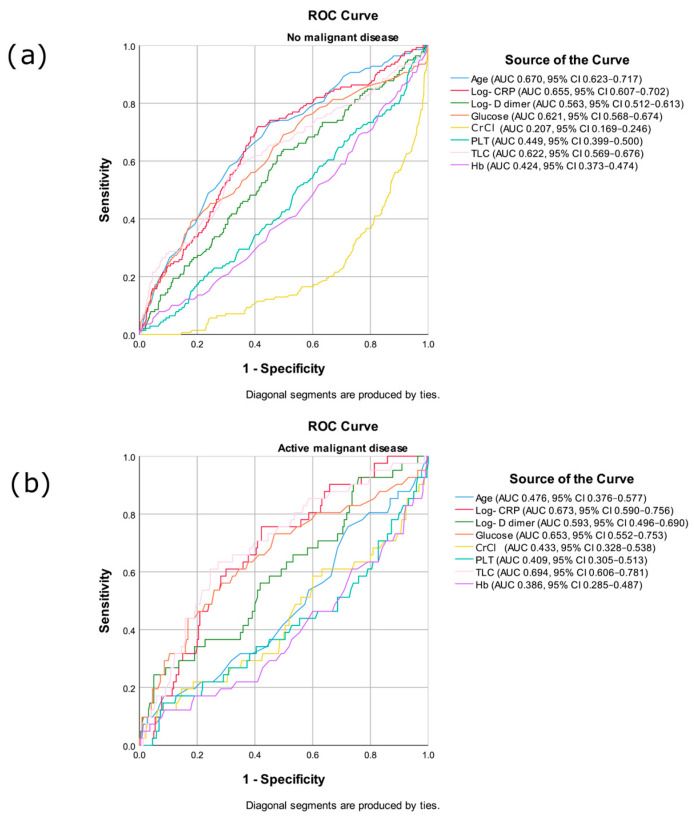
Receiver operating characteristic (ROC) analyses of clinical variables stratified by malignancy status. (**a**) ROC curves for patients without malignancy. (**b**) ROC curves for patients with active malignancy. Variables shown include age, CRP, D-dimer, glucose, CrCl, platelet count, total leukocyte count, and hemoglobin. AUC values with 95% confidence intervals are provided in the figure.

To further explore the discriminatory performance of individual biomarkers, ROC analyses were performed separately in patients with and without malignancy (Figure 3).

In the non-malignant group, age (AUC 0.670, 95% CI 0.623–0.717), CRP (AUC 0.655, 95% CI 0.607–0.702), glucose (AUC 0.621, 95% CI 0.568–0.674), and leukocyte count (AUC 0.622, 95% CI 0.569–0.676) demonstrated moderate discrimination. In contrast, in the malignant group, CRP (AUC 0.673, 95% CI 0.590–0.756), glucose (AUC 0.653, 95% CI 0.552–0.753), and leukocyte count (AUC 0.694, 95% CI 0.606–0.781) retained discriminatory ability, whereas age and CrCl lost predictive value (AUC 0.476 and 0.433, respectively).

## 4. Discussion

Two main findings emerged from the present study. First, patients with cancer-associated PE had a significantly higher in-hospital mortality rate than patients without cancer. Second, cancer patients presented with more severe PE at diagnosis, confirming findings from previous studies. Beyond these observations, our study examined routinely available laboratory parameters and their prognostic performance in patients with and without active malignancy and specifically tested whether their predictive value for in-hospital mortality differs between the two cohorts in a real-world clinical practice setting.

In the univariate analysis of a cohort, a range of parameters was identified as being associated with in-hospital mortality, including age, CRP, BNP, D-dimer, glucose, CrCl, PLT, Hb, PTE risk category, and malignancy. Based on these findings, a multivariable model was constructed in which CRP, glucose, CrCl, PLT, PTE risk category, and malignancy emerged as independent predictors (BNP was excluded due to a substantial proportion of missing data). These biomarkers capture different dimensions of the physiological response to an acute embolic event and collectively enable a comprehensive assessment of disease severity.

Elevated CRP reflects systemic inflammation and stress response [15,16,17]. Lower platelet counts may reflect consumptive coagulopathy and increased thrombus burden [18]. Glucose reflects stress-induced hyperglycemia and endothelial dysfunction (8,20,21), while reduced eGFR signals impaired cardiorenal reserve and multi-organ vulnerability [9,19,20,21,22]. The predictive value of these markers (including PTE risk category) in PE patients is therefore expected and has been consistently confirmed in previous studies. Interestingly, although numerous studies have demonstrated that troponin is a reliable marker of short-term prognosis in acute PE, as confirmed by the current AHA/ACC guidelines, in our cohort, troponin I did not show a significant association with in-hospital mortality [23].

### 4.1. Impact of Malignancy as a Central Determinant of Outcome

Malignancy emerged as an important independent determinant of outcome. Its impact may reflect a complex interplay of hypercoagulability, tumor burden, aggressive disease biology, systemic inflammation, and a higher burden of comorbidities, along with frequent accompanying factors such as immobility, frailty, and malnutrition. In our cohort, patients with active malignancy had approximately 1.7–fold higher odds of in-hospital mortality, and malignancy remained an independent predictor in multivariable analysis, consistent with numerous prior studies identifying it as an important risk factor for adverse PE outcomes [13,24,25,26,27]. However, the question arises whether malignancy is merely a predictor in this cohort or exerts a broader influence.

### 4.2. Comparative Clinical and Biological Profiles: Malignant vs. Non-Malignant PE

Direct comparison between patients with malignant and non-malignant PE revealed significant differences. In particular, we focused on those laboratory parameters that emerged as significant predictors of in-hospital mortality in the univariate regression analysis. Patients with malignancy were significantly older, likely reflecting the increased incidence of both cancer and venous thromboembolism in older populations [28,29].

Patients with malignancy exhibited significantly higher CRP levels and TLC, consistent with malignancy-associated inflammatory processes. Cancer patients frequently exhibit chronic inflammation related to both the tumor and its treatment. Elevated values may reflect a paraneoplastic inflammatory response, driven by tumor-derived cytokines such as TNF–α and IL–6, which stimulate bone marrow activity and hepatic CRP synthesis [30,31,32].

Lower Hb and CrCl in the malignant group could indicate a more pronounced systemic and multi-organ impact of cancer. Although borderline significant in this cohort, reduced CrCl may result from nephrotoxic effects of chemotherapy, paraneoplastic glomerulopathies, or a higher prevalence of chronic kidney disease in older, comorbid populations. Lower Hb levels may reflect anemia of chronic disease, as well as the effects of chemotherapy or radiotherapy. 

Elevated platelet counts in patients with malignancy likely reflect tumor-driven inflammation and reactive thrombocytosis, which correlate with disease progression and enhanced thrombotic activity [33]. Given the borderline *p*-value, we cannot assert a statistically robust difference, yet the tendency toward higher values in malignancy is evident. D-dimer levels were significantly higher in cancer patients. This elevation may reflect the prothrombotic milieu characteristic of cancer, where tumor cells are thought to promote continuous thrombin generation and fibrin formation through tissue factor expression and cytokine-mediated pathways, thereby contributing to the greater thrombotic burden observed in malignancy [34]. The shift toward higher ESC/PTE risk categories in patients with active malignancy likely reflects both a methodological effect (malignancy is a scored item within PESI, which contributes directly to the composite ESC classification) and true clinical severity (older age, comorbidity and adverse laboratory/clinical features); therefore, risk-stratification in cancer patients should be interpreted with caution and supplemented by cancer-specific context.

Overall, in-hospital mortality was nearly twice as high in the malignant cohort. Among deaths, a smaller proportion of fatalities in the malignancy group was directly attributable to PE compared with the non-malignancy group, a pattern also observed in large PE registries, where cancer-related mortality frequently predominates over PE as the proximate cause [35]. No significant differences were observed between groups in other parameters (glucose, BNP).

### 4.3. Analysis of Predictors of In-Hospital Mortality: Malignant vs. Non-Malignant PE

Comparative analysis of prognostic factors between patients with PE with and without malignancy reveals clearly divergent risk patterns. In the non-malignant population, univariate analysis identified multiple parameters (age, CRP, BNP, D-dimer, glucose, CrCl, Hb, TLC, and ESC risk category) as significant predictors of in-hospital mortality. In contrast, in patients with malignancy, some of these parameters lost statistical significance, suggesting that the presence of malignancy substantially alters the interpretation of conventional prognostic markers.

This pattern may reflect a masking effect of malignancy, whereby cancer-related inflammation, organ dysfunction, and treatment-related factors reduce the discriminatory value of standard PE biomarkers.

#### 4.3.1. Loss of Predictive Power of Traditional Biomarkers in Malignancy

One example is the loss of the prognostic significance of age in patients with malignancy. This may be explained by the dominance of tumor biology over chronological factors, with disease stage, metastatic burden, and overall tumor load exerting a greater influence on short-term outcomes than age itself [36].

A similar pattern was observed for TLC. As mentioned before, underlying malignancy, systemic inflammation, and oncologic therapy may reduce their specificity as a marker of PE severity.

Although CrCl and hemoglobin were significant predictors in non-malignant patients, their prognostic value was lost in the malignant group, consistent with a significant interaction between CrCl and malignancy status. In cancer patients, renal dysfunction and anemia are frequently present at baseline, which may limit their ability to discriminate outcomes. Additionally, CrCl estimation may be less reliable in cancer patients due to sarcopenia and reduced creatinine production, leading to potential overestimation of renal function with creatinine-based equations. In this context, alternative markers such as cystatin C may provide a more accurate assessment [37].

An additional notable finding is the role of platelet count, which emerged as a significant predictor of mortality exclusively in the malignant group, despite no significant difference in mean values between groups (there is even a trend toward higher values in patients with malignancy, *p* = 0.050). While thrombocytosis is often associated with poor prognosis in cancer due to its role in tumor progression and hypercoagulability, our results could indicate that relative reductions in platelet count, even within the normal laboratory range and in the absence of overt thrombocytopenia, are associated with increased mortality [38,39]. Malignancy is known to profoundly influence platelet activation, consumption, and thromboinflammatory signaling, and, therefore, part of the prognostic effect attributed to platelet count may reflect cancer-related systemic processes rather than an entirely independent mechanism. This suggests that relative thrombocytopenia may be a marker of reduced hematologic reserve, advanced disease, or treatment-related toxicity in cancer-associated PE. There is a limited body of literature addressing this phenomenon; a 2024 study demonstrated that patients with pulmonary embolism and markedly low platelet counts have higher mortality rates. However, our findings indicate that platelet count is a predictor only within the subgroup of PE patients with malignancy [40].

#### 4.3.2. Stable and Universal Predictors Across Subgroups

Despite these differences, certain biomarkers retain prognostic value across both groups. BNP, as an indicator of right ventricular strain and the immediate mechanism of hemodynamic collapse, remains relevant regardless of malignancy status (univariate analysis), in line with the existing literature [28,41,42]. D-dimer, reflecting overall thrombotic burden and coagulation activation, maintains a central role in risk assessment [41,43]. Similarly, glucose remains a strong predictor in both populations, supporting the concept that stress-induced hyperglycemia represents a universal marker of acute illness severity (although the predictive value varied according to malignancy) [9,19,20]. The results also demonstrated that CRP is a predictor of mortality in both groups, which is consistent with the literature emphasizing that CRP may be useful in the context of oncological PE [44,45].

Multivariate analysis showed that CRP and glucose retained prognostic value across both groups, whereas in the non-malignant population, CrCl was also independently associated with mortality. This pattern is consistent with the previously described findings. Importantly, ESC risk category was an independent predictor only in the non-malignant group. This may potentially be explained by the construction of the risk stratification algorithm itself (as mentioned before). As a result, the incremental prognostic contribution of risk category may be attenuated in the malignant cohort.

In non-malignant patients, there is a progressive and statistically significant increase in risk across higher CRP quartiles (Q3 and Q4 vs. Q1), reflecting the direct, deleterious impact of heightened systemic inflammation on the host. By contrast, among patients with active malignancy, the increases in risk across CRP quartiles do not reach statistical significance, which may reflect a degree of physiological adaptation to chronic, tumour-induced low-grade inflammation. Glucose shows the opposite pattern: in non-malignant patients, quartile-based increases in glucose produce only a modest, non-significant rise in risk, whereas in the oncological cohort, risk rises sharply and becomes highly significant only in the highest quartile (Q4 vs. Q1: OR = 3.321, *p* = 0.005). This isolated jump may reflect the existence of a metabolic threshold associated with severe insulin resistance, but alternative explanations cannot be excluded.

Moreover, significant interactions between malignancy and CrCl, age, TLC, and glucose suggest that the effect of these variables on mortality is not uniform but may depend on malignancy status. Stratified ROC analyses supported these findings: age and CrCl were strong predictors in patients without malignancy but lost discriminatory power in those with active malignancy. Conversely, CRP, glucose, and leukocyte count maintained or even improved predictive performance in the malignant subgroup. In this context, the loss of prognostic significance of CrCl in cancer patients does not necessarily imply biological irrelevance, but may reflect reduced discriminatory capacity in the setting of baseline homeostatic imbalance. Glucose, although it significantly interacts with malignancy, appears relatively independent of these chronic influences and retains stable prognostic value across both populations. Taken together, these findings indicate that malignancy acts not only as an independent risk factor but also as a potential modifier of biomarker performance in acute PE. These findings suggest that cancer-associated PE may represent more than just a higher-risk form of PE, potentially constituting a subgroup with unique prognostic characteristics.

### 4.4. Clinical Implications

Our findings suggest that conventional risk stratification models developed in unselected PE populations may not be directly applicable to patients with malignancy. Biomarkers such as CrCl should be interpreted with caution in oncological patients, as baseline abnormalities may reduce their discriminatory capacity. In contrast, glucose remained independently associated with mortality in both subgroups, although effect modification was observed. These results support the need for malignancy-adapted risk stratification approaches that integrate both cancer-related and PE-related factors to improve prognostic accuracy.

### 4.5. Study Limitations

This study has several limitations. First, its retrospective design introduces the possibility of residual confounding and limits causal inference. Second, missing data for certain variables, particularly BNP, may have influenced the multivariable models. Third, although oncological data (including primary tumour site, recent anticancer therapy and metastatic status) were available and are presented here (Supplementary Materials) for descriptive purposes, these variables were not incorporated into the present multivariable analyses because inclusion of detailed oncologic covariates was beyond the predefined scope of this study. Finally, only in-hospital outcomes were assessed, without long-term follow-up. These limitations should be considered when interpreting the results.

## 5. Conclusions

This large, real-world cohort study suggests that active malignancy may act as an independent predictor of in-hospital mortality in acute PE and appears to be associated with a more severe clinical and laboratory profile at presentation. Beyond its potential role as an independent risk factor, our findings suggest that malignancy may also modify the prognostic performance of standard biomarkers: while CRP and glucose appeared to retain independent predictive value across both subgroups, CrCl seemed to lose discriminatory capacity in cancer patients, a finding further supported by significant interaction effects and stratified ROC analyses. Notably, although additional parameters such as age, total leukocyte count, and hemoglobin did not remain independent predictors in the multivariable model, their significant interactions with malignancy status suggest that their prognostic contribution may be differentially modulated by the presence of cancer. These observations raise the possibility that cancer-associated PE behaves differently from non-malignant PE, potentially representing a subgroup with unique prognostic patterns rather than simply a higher-risk presentation. As these findings are based on observational data, they should be interpreted as associations rather than causal relationships. Consequently, standard risk stratification tools developed in unselected populations may require careful re-interpretation when applied to oncological patients. Prospective studies designed to validate malignancy-adapted prognostic frameworks are warranted to improve risk assessment and guide clinical decision-making in this challenging population.

## Figures and Tables

**Table 1:** Baseline characteristics of the study population.

Parameter	*N* (Patients)	Value
Male, *N*, %	2803	1320, 47.09
Age (years), mean ± SD	2803	64.78 ± 15.63
Active malignancy, *N*, %	2803	393, 14.02
In-hospital mortality, *N*, %	2803	311, 11.10
PE-related in-hospital mortality, *N*, %	311	173, 55.63
CRP (mg/L), median (25th–75th)	2691	42.40 (15.50–95.30)
BNP (pg/mL), median (25th–75th)	882	176.85 (57.30–484.50)
Troponin I (ng/mL), median (25th–75th)	1139	0.08 (0.01–0.53)
D-dimer (ng/mL), median (25th–75th)	2597	4260.00 (2228.50–8972.50)
Glucose (mmol/L), mean ± SD	2718	7.89 ± 7.62
CrCl (mL/min), mean ± SD	2181	76.06 ± 35.96
PLT (×10^9^/L), mean ± SD	2729	243.10 ± 102.55
TLC (×10^9^/L), mean ± SD	2779	11.02 ± 6.50
Neutrophils (×10^9^/L), median (25th–75th)	1720	7.90 (5.69–11.40)
Lymphocytes (×10^9^/L), median (25th–75th)	1538	1.62 (1.15–2.22)
Hemoglobin (g/L), mean ± SD	2778	129.43 ± 21.63
Hematocrit (%), mean ± SD	2668	39.19 ± 7.65

Abbreviations: SD, standard deviation; CRP, C-reactive protein; BNP, B-type natriuretic peptide; CrCl, creatinine clearance; PLT, platelet count; TLC, total leukocyte count.

**Table 2:** Univariate and multivariable logistic regression analysis of predictors of in-hospital mortality.

Parameter	Univariate Analysis OR (95% CI, *p*)	Multivariable Analysis OR (95% CI, *p*)
Sex (Male vs. Female)	0.809 (0.638–1.027, 0.082)	–
Age (years)	1.035 (1.026–1.045, <0.001)	–
Active malignancy (yes vs. no)	1.743 (1.295–2.346, <0.001)	1.660 (1.089–2.532, 0.019)
Log-CRP (mg/L)	1.779 (1.570–2.016, <0.001)	1.545 (1.308–1.826, <0.001)
Log-BNP (pg/mL)	1.604 (1.355–1.898, <0.001)	–
Log-Troponin I (ng/mL)	1.093 (0.957–1.249, 0.188)	–
Log-D-dimer (ng/mL)	1.276 (1.141–1.428, <0.001)	–
Glucose (mmol/L)	1.016 (1.001–1.031, 0.034)	1.018 (1.005–1.031, 0.007)
CrCl (mL/min)	0.970 (0.965–0.976, <0.001)	0.975 (0.969–0.981, <0.001)
PLT (×10^9^/L)	0.998 (0.997–0.999, 0.004)	0.998 (0.996–1.000, 0.021)
TLC (×10^9^/L)	1.057 (1.038–1.077, <0.001)	–
Neutrophils (×10^9^/L)	1.002 (0.998–1.006, 0.356)	–
Lymphocytes (×10^9^/L)	0.992 (0.930–1.058, 0.801)	–
Hemoglobin (g/L)	0.990 (0.985–0.996, <0.001)	–
Hematocrit (%)	0.986 (0.971–1.002, 0.084)	–
PTE risk category (reference Low Risk)		
Intermediate Low Risk	1.785 (1.057–3.014, 0.030)	1.652 (0.865–3.153, 0.128)
Intermediate High Risk	4.350 (2.760–6.856, <0.001)	3.055 (1.745–5.348, <0.001)
High Risk	16.286 (10.359–25.603, <0.001)	7.966 (4.430–14.323, <0.001)

Multivariable logistic regression included *N* = 1878 patients with complete data for all variables. BNP was excluded from the multivariable model due to a high proportion of missing values. Abbreviations: OR, odds ratio; CI, confidence interval; CRP, C-reactive protein; BNP, B-type natriuretic peptide; CrCl, creatinine clearance; PLT, platelet count; TLC, total leukocyte count; Log-, logarithmically transformed variable.

**Table 3:** Comparison of baseline characteristics between patients with and without active malignancy.

Parameter	No Malignancy	Active Malignancy	*p*
CRP (mg/L), median (25th–75th)	39.25 (14.80–89.33)	63.50 (24.00–127.15)	<0.001
BNP (pg/mL), median (25th–75th)	176.85 (57.34–489.00)	177.15 (56.20–476.00)	0.990
Troponin I (ng/mL)	0.08 (0.01–0.52)	0.08 (0.01–0.65)	0.735
D-dimer (ng/mL), median (25th–75th)	4220.00 (2187.00–8620.00)	4879.00 (2500.00–10,058.50)	0.016
Glucose (mmol/L), mean ± SD	7.96 ± 8.12	7.47 ± 3.16	0.238
CrCl (mL/min), mean ± SD	76.65 ± 36.18	72.23 ± 34.38	0.051
TLC (×10^9^/L), mean ± SD	10.86 ± 5.30	12.02 ± 11.32	0.048
PLT (×10^9^/L), mean ± SD	241.25 ± 98.62	254.18 ± 123.21	0.050
Neutrophils (×10^9^/L)	7.90 (5.68–11.17)	8.40 (5.98–13.32)	0.103
Lymphocytes (×10^9^/L)	1.65 (1.20–2.23)	1.50 (1.00–2.12)	0.014
Hemoglobin (g/L), mean ± SD	13.13 ± 2.10	11.76 ± 2.18	<0.001
Hematocrit (%), mean ± SD	39.75 ± 7.61	35.77 ± 6.99	<0.001
PTE risk category			
Low Risk, *N*, %	725, 30.08	70, 17.81	<0.001
Intermediate Low Risk, *N*, %	595, 24.69	108, 27.48	
Intermediate High Risk, *N*, %	741, 30.75	130, 33.08	
High Risk, *N*, %	349, 14.48	85, 21.63	

Abbreviations: SD, standard deviation; CRP, C-reactive protein; BNP, B-type natriuretic peptide; CrCl, creatinine clearance; TLC, total leukocyte count; PLT, platelet count.

**Table 4:** Univariate and multivariable logistic regression analysis of predictors of in-hospital mortality according to malignancy status.

Parameter		Univariate Analysis OR (95% CI, *p*)	Multivariable Analysis OR (95% CI, *p*)
Age (years)	Active malignancy	1.013 (0.990–1.037, 0.275)	–
	No malignancy	1.039 (1.029–1.050, <0.001)	–
Log-CRP (mg/L)	Active malignancy	1.497 (1.141–1.963, 0.004)	1.911 (1.275–2.865, 0.002)
	No malignancy	1.819 (1.580–2.093, <0.001)	1.449 (1.204–1.743, <0.001)
Log-BNP (pg/mL)	Active malignancy	1.510 (1.078–2.115, 0.016)	–
	No malignancy	1.659 (1.361–2.022, <0.001)	–
Log-D-dimer (ng/mL)	Active malignancy	1.520 (1.161–1.990, 0.002)	–
	No malignancy	1.212 (1.073–1.369, 0.002)	–
Glucose (mmol/L)	Active malignancy	1.135 (1.053–1.222, 0.001)	1.192 (1.075–1.322, 0.001)
	No malignancy	1.013 (1.000–1.026, 0.050)	1.014 (1.001–1.028, 0.034)
CrCl (mL/min)	Active malignancy	0.994 (0.985–1.004, 0.238)	–
	No malignancy	0.963 (0.956–0.969, <0.001)	0.964 (0.957–0.972, <0.001)
PLT (×10^9^/L)	Active malignancy	0.996 (0.994–0.999, 0.006)	–
	No malignancy	0.999 (0.997–1.000, 0.074)	–
TLC (×10^9^/L)	Active malignancy	1.015 (0.995–1.035, 0.154)	–
	No malignancy	1.080 (1.055–1.106, <0.001)	–
Hemoglobin (g/L)	Active malignancy	0.995 (0.983–1.008, 0.461)	–
	No malignancy	0.991 (0.985–0.998, 0.006)	–
PTE risk category (reference Low Risk)			
Intermediate Low Risk	Active malignancy	1.320 (0.382–4.561, 0.661)	–
	No malignancy	1.806 (1.011–3.227, 0.046)	1.606 (0.754–3.418, 0.219)
Intermediate High Risk	Active malignancy	4.125 (1.377–12.354, 0.011)	–
	No malignancy	4.147 (2.509–6.855, <0.001)	3.035 (1.602–5.750, 0.001)
High Risk	Active malignancy	7.681 (2.537–23.254. <0.001)	–
	No malignancy	18.24 (11.101–29.964, <0.001)	9.210 (4.712–18.001, <0.001)

Multivariable logistic regression included 261 patients with malignancy and 1617 patients without. BNP was excluded from the multivariable model due to a high proportion of missing values. Abbreviations: OR, odds ratio; CI, confidence interval; CRP, C-reactive protein; BNP, B-type natriuretic peptide; CrCl, creatinine clearance; PLT, platelet count; TLC, total leukocyte count; Log–, logarithmically transformed variable.

## Data Availability

Data will be available upon reasonable request.

## Supplementary Materials

The following supporting information can be downloaded at: https://www.mdpi.com/article/10.3390/diagnostics16132130/s1: Table S1. Clinical characteristics of the malignant subgroup (N = 393).

## Abbreviations

The following abbreviations are used in this manuscript:

PE	Pulmonary embolism
REPER	Regional Pulmonary Embolism Registry
PESI	Pulmonary Embolism Severity Index
sPESI	Simplified Pulmonary Embolism Severity Index
VTE	Venous thromboembolism
MDCT-PA	Multi-detector computed tomography pulmonary angiography
CRP	C-reactive protein
BNP	B-type natriuretic peptide
eGFR	estimated glomerular filtration
CrCl	creatinine clearance
PLT	Platelet count
TLC	Total leukocyte count
Hb	Hemoglobin
OR	Odds ratio
CI	Confidence interval
IQR	Interquartile range
SD	Standard deviation
