# Supplementary material for: Malignancy as a Predictor and Potential Modifier of Laboratory Biomarker Prognostic Value in Acute Pulmonary Embolism
Source: Diagnostics (Basel). 2026 Jul 7;16(13):2130. doi: 10.3390/diagnostics16132130 (PMC13361538; doi:10.3390/diagnostics16132130)
Supplement: Supplementary file 1 [file diagnostics-16-02130-s001.zip › diagnostics-4384216-supplementary.pdf]

Characteristic	*n*	%
**Pulmonary embolism as first manifestation**	117	29.77
**Chemotherapy within 7 days**	80	20.36
**Radiotherapy within 7 days**	35	8.91
**Metastatic disease**	111	28.24
**Primary tumor site**		
**Gastrointestinal (total)**	83	21.12
**Colorectal**	36	9.16
**Gastric**	13	3.31
**Esophageal**	2	0.51
**Hepatic**	12	3.05
**Pancreatic**	11	2.80
**Other GI**	9	2.29
**Urogenital (total)**	82	20.87
**Renal/Bladder**	30	7.63
**Prostate**	20	5.09
**Gynecological**	32	8.14
**Lung**	71	18.07
**Breast**	39	9.92
**Hematological**	27	6.87
**Brain**	14	3.56
